# Analgesic Use in Patients during Cardio-Pulmonary Resuscitation

**DOI:** 10.3390/ijerph20043654

**Published:** 2023-02-18

**Authors:** Sebastian Dąbrowski, Sandra Lange, Andrzej Basiński

**Affiliations:** 1 Department of Medical Rescue, Faculty of Health Sciences with the Institute of Maritime and Tropical Medicine, Medical University of Gdańsk, Dębinki 7, 80-211 Gdańsk, Poland; 2 Department of Internal and Pediatric Nursing, Medical University of Gdańsk, Dębinki 7, 80-211 Gdańsk, Poland

**Keywords:** resuscitation, awareness, cardiac arrest, memory, analgosedation, trauma, fractures

## Abstract

Introduction: Cardiopulmonary resuscitation-induced consciousness is a newly recognized phenomenon with an increasing incidence. A return of consciousness during cardiopulmonary resuscitation affects up to 0.9% of cases. Patients may also experience physical pain associated with chest compressions, as most victims of cardiac arrest who are subjected to resuscitative efforts sustain ribs or sternum fractures. Methods: A rapid review was carried out from August 2021 to December 2022. Results: Thirty-two articles were included in the rapid review. Of these, eleven studies focused on the return of consciousness during CPR, and twenty-one on CPR-induced chest injuries. Conclusion: A small number of studies that have dealt with the return of consciousness associated with cardiopulmonary resuscitation made it hard to clearly determine how often this occurs. There were more studies that dealt with chest trauma during resuscitation, but no study considered the use of analgesics. Of note, there was no standardized therapeutic approach as far as the use of analgesics and/or sedatives was considered. This is probably due to the lack of guidelines for analgesic management during cardiopulmonary resuscitation and peri-resuscitative period.

## 1. Introduction

Cardiopulmonary resuscitation (CPR) is a priority for the survival of patients with sudden cardiac arrest (SCA). Chest compressions are the key component to ensure the flow of oxygenated blood to the brain and heart [1]. Thoracic fractures are the most common injuries suffered when the chest is compressed (a third of patients suffer rib fractures and a fifth suffer sternum fractures) [2]. Cardiac arrest is always associated with a loss of consciousness. CPR-induced consciousness is a newly recognized phenomenon with an increasing prevalence. It can be associated with early, high-quality cardiopulmonary resuscitation, using mechanical devices and performed by qualified personnel. The return of consciousness during CPR affects 0.23% to 0.9% of cases, and is more common in younger, healthier patients [3,4,5,6,7]. Patients may experience physical pain associated with CPR. Pain relief can often be overlooked during intensive resuscitation, especially in intubated patients [6,7]. There is a lack of specific management in the international perspective. A Netherlands guide on the management of patients who are agitated or suffering from pain during chest compressions suggests the use of fentanyl and midazolam [8]. In the Olaussen et al. review, the return of consciousness during CPR was very variable. The management of such cases included physical restraint and the administration of benzodiazepines and/or opiates [9]. A study by Chao et al. showed that patients intubated during resuscitation are probably receiving inadequate analgesia. The inadequacy appears to relate to the timing and repetition of administration rather than the dose. Patients who were transferred early to the intensive care unit were more likely to receive analgesia [10]. The best hospital care for patients with a return of spontaneous circulation (ROSC) after cardiac arrest is not fully known, but there is growing interest in identifying and optimizing practices that can improve survival outcomes [11].

### Aim

A rapid review was conducted to determine whether analgesic treatment or sedation was used in patients who showed signs of consciousness during resuscitation or may have had a rib or sternum fracture.

## 2. Methods

### 2.1. Study Design

A rapid review was carried out from August 2021 to December 2022.

### 2.2. Review Questions

To identify important aspects of pain management in patients during CPR, we developed research questions that clearly define the population, concept, and context (PCC) of the rapid review.

1. How often does the return of consciousness occur during CPR?
2. What is the incidence of chest injuries in patients during CPR?
3. What painkillers and sedatives are used to improve treatment?

### 2.3. Definition of a Rapid Review

A formal definition for a rapid review does not exist. As such, we used the following working definition: “a rapid review is a type of knowledge synthesis in which components of the systematic review process are simplified or omitted to produce information in a short period of time’’ [12].

### 2.4. Search Strategy

Three authors systematically searched the following databases: PubMed, Web of Science, and Cochrane Library. In addition, to identify studies that were relevant to the review, we checked publication references. The following keywords were used: “Emergency Care”, “ICU”, “Cardiopulmonary resuscitation”, “Cardiac arrest”, “Chest compression”, “Chest injuries”, “Rib fractures”, “Sternum fracture”, and “CPR induced consciousness”. Keywords were entered along with their combinations using AND or OR. All publications were analyzed by title and abstract to exclude inaccurate entries. Any discrepancy was resolved through discussion with the four researchers, and at the end of the selection process, full agreement was reached on the articles to be included. Data including author (first), target, participants, interventions, results, and findings were extracted from all eligible studies. The number of articles found in each search test was limited to the 2010–2022 surveys. The initial search was carried out from the beginning of August 2021 to May 2022, and the final search was carried out on May 31 2022. In order to identify the relevant studies, we used the Population–Concept–Context (PCC) framework recommended by the Joanna Briggs Institute (JBI) [13]. Strict inclusion and exclusion criteria were applied (Table 1). Reviews are considered eligible if all of the following criteria are met.

### 2.5. Study Selection

In line with the PCC framework, our scope review included data from studies of adult ICU patients (>18 years of age) (P) diagnosed with a chest injury or with return of consciousness (C) that occurred during ongoing CPR (C). We excluded studies in which the participants were children (<18 years old), non-ICU patients, patients with undiagnosed chest damage, and recurrence of consciousness after cardiac arrest. We also excluded publications in a language other than English and articles that could not be accessed in the full version.

### 2.6. Data Extraction

The data extraction, referred to in the rapid review as the “data plot”, was undertaken independently by the three reviewers. Information obtained from the included studies for awareness assessment included the following: first author’s name and surname, year of publication, place of resuscitation, type of chest compressions, use of analgesia and/or sedation, whether CPR was successful, and whether there were any signs of recovery. Information obtained from the included studies for the assessment of chest injuries included the following: first author’s name and surname, year of publication, number of participants, place of resuscitation, population, country, age, gender, type of chest compressions, use of analgesia and/or sedation, frequency of sternum and rib fractures, and how the injury was diagnosed. The authors performed an extraction using Microsoft Excel 2021.

### 2.7. Assessment of the Quality of Studies Included in the Studies

The purpose of this rapid review was to compile the published information about the altered consciousness and the chest trauma that patients experienced during CPR. Since we chose to conduct this type of review, we did not critically evaluate the individual sources of evidence. In the case of a scope review, it is allowed to review the current evidence without taking into account the methodological assessment of the included studies.

## 3. Results

**Figure 1:**
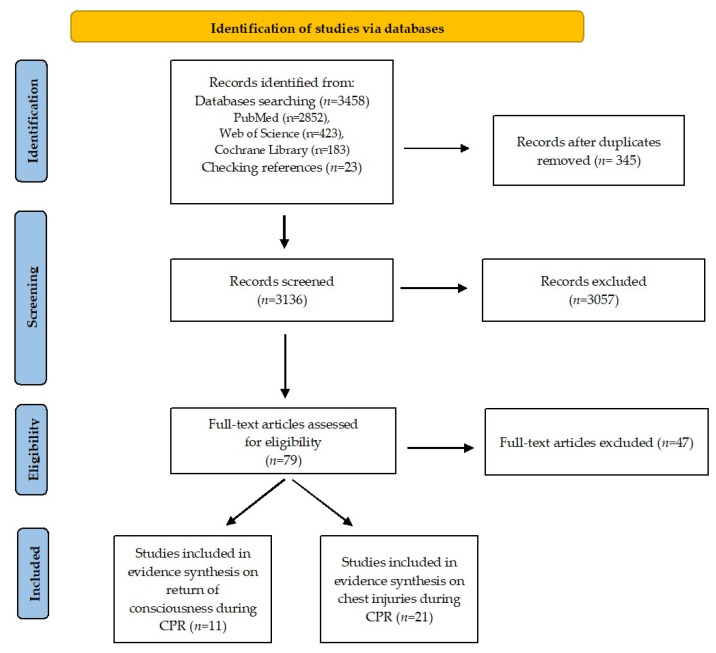
PRISMA flow diagram. Reprinted/adapted with permission from Ref. [14]. 2020, Matthew J Page [14].

A total of 3458 records was initially obtained from the databases: PubMed—2852, Web of Science—423, and Cochrane Library—183, with an additional 23 records after reference checking. After discarding duplicates and selecting titles and abstracts, 3402 were excluded, leaving 79 articles that were analyzed. Of these, 47 were excluded for failing to meet the inclusion criteria. A total of 32 articles met the inclusion criteria. The results are presented in Figure 1.

### 3.1. Return of Consciousness during CPR

Chest compressions cause slight but critical blood flow to the brain, and sometimes cerebral perfusion pressure is sufficient to restore the patient’s consciousness, Table 2 [4,7,15,16,17,18,19,20,21,22,23]. CPR-induced awareness (CPR-IC) can be defined as the presence of clinical signs of cerebral perfusion during CPR that do not occur when CPR is interrupted [15]. Some authors divide signs of CPR-IC into two groups. In the first one, there are visible signs of consciousness such as combativeness, groaning, and eye opening. In the second, there are no external signs of consciousness, but a perception of lucidity with visual and auditory awareness and recall is present [6]. The use of an automatic cardiopulmonary resuscitation device was clearly part of successful resuscitation due to its ability to provide consistent, continuous, high-quality CPR while allowing rescuers [6] to perform other essential tasks. During chest compressions with LUCAS device, the patient was provided with adequate perfusion to regain consciousness [4,7,15,18,20,22]. During manual chest compressions, returns of consciousness also occurred [4,15,17,18,19,20,21,22]. In the studies by authors Olaussen et al. and Grandi et al., the administration of analgesics and sedatives during resuscitation was demonstrated [18,22]. In contrast, in the studies by Rice et al., Pound et al., and Ulrichs et al., only sedative drugs were used [7,16,21]. In the study by Pinot et al., the researchers did not use sedation because they did not know what effect this would have on the success of CPR, they only used direct restraints; however, the authors suggest ketamine as the most beneficial drug [4].

### 3.2. Chest Injuries during CPR

Cardiopulmonary resuscitation can cause chest injuries—Table 3 [24,25,26,27,28,29,30,31,32,33,34,35,36,37,38,39,40,41,42,43,44]. The incidence of injury is variable and can reach up to 100%, as demonstrated in a study by Jang SJ et al. Differences in the number of injuries diagnosed may be related to the sensitivity of specific diagnostic tests. [39]. Injuries were most diagnosed by CT scans or by performing autopsies on non-survivors of CPR. In the Zaidi et al. study, X-ray had a low sensitivity in detecting complications after CPR compared with CT scans [40]. Most studies used manual chest compressions. Despite the high incidence of chest injury, none of the studies reported whether analgesia or sedation was used during CPR. Older age was found to be an independent risk factor for rib/sternal fracture in studies by Kashiwagi et al., Yamaguchi et al., Krajl et al., and Setälä et al. [28,29,31,35]. The impact of using a mechanical device during CPR is not clear. Studies by Smekal et al. and Freiberg et al., comparing manual vs. mechanical chest compressions showed that rib fractures were more common after the use of a mechanical device [35,36]. In contrast, in the study by Kralij et al. and Canakci et al., no significant difference was observed in terms of complications in patients who received mechanical compression compared to those who received only manual compression [29,43]. The implementation of the new guidelines in 2010 resulted in an increase in the rate of complications during CRP, with a concomitant increase in survival rates [33]. In the study by Kralj et al., the change in guidelines from 2005 to 2010 was also found to be a contributory factor in the occurrence of thoracic skeletal injuries [29]. Hallevuo et al. showed that deeper chest compressions were associated with more injuries [24]. On the other hand, Yamaguchi et al. found only a small effect of the updated guidelines on the incidence of CPR-related injuries [31]. Yusufloglu et al. found no difference in the incidence of CPR-related injuries when comparing CPR performed according to the AHA 2010 vs. ERC 2015 guidelines [34].

## 4. Discussion

With the continually evolving guidelines for CPR, and hence, the improving qualitative resuscitation efforts, rescuers appear to be dealing with an increasing number of patients who may be experiencing a variety of cognitive processes during this time. In addition, given the known fact that CPR often causes damage to the skeleton and internal organs, it seems appropriate to start a discussion on the use of sedatives and analgesics in this type of patients.

### 4.1. Return of Consciousness during CPR

There are reports of patients who, although far from physiological perfusion values, showed signs of CPR-induced consciousness. The etiology of this phenomenon does not seem to be well-understood, as there is no measurable brain function within seconds after cardiac standstill and yet some patients maintained awareness a few minutes into cardiac arrest [6]. Although there is no universally accepted definition, CPR-IC can be characterized by the presence of spontaneous eye opening, arm, leg, or trunk movements, speaking, following commands, feeling pain, or even resisting CPR during cardiac arrest [4]. Some authors emphasize though that consciousness may be present in some patients but it may be clinically undetectable. [6]. The estimates from observational studies are that CPR-induced consciousness occurred in 0.23% to 0.9% of all CPR efforts [35]. The apparent increasing incidence of this phenomenon creates new challenges for CPR providers and may even influence the outcome of CPR in patients that are comparative. In addition, there are a growing number of reports of patients who remember what happened during CPR, which can have serious psychological consequences for survivors [6]. A significant proportion of patients may develop cognitive sequelae after resuscitation, such as post-resuscitation post-traumatic stress disorder (PTSD) and long-term memory [7]. The use of sedative and analgesic drugs has greater potential to provide a better quality of care, although the evidence of their safety is lacking. The choice of the drug used must take into account not only its sedative and analgesic effects, but also, or even more importantly, its hemodynamic properties [6].

### 4.2. Chest Injuries during CPR

The incidence of chest injuries during CRP is high and these are usually multiple injuries [25,27,29,33,34,36,42,45]. Thoracic wall injuries are associated with poorer hospital outcomes, such as fewer ventilator-free days and higher mortality [43]. Additionally, it has been noted that rib fractures caused by successful resuscitation are associated with an increased incidence of pneumonia [45]. Fractures of the ribs or sternum lead to difficulty breathing, shallow breathing and the presence of pain. However, the clinical symptoms of pain, the measurement of pain severity, and the use of pain treatment were not included in any of the studies. Therefore, we were not able to obtain an answer to our research question. However, we would like to hypothesize for future research that the use of analgesic treatment during or in the immediate post-resuscitation period may have beneficial effects. Should, in the 21st century, the fear of adverse hemodynamics preclude medical professionals from using sedatives or analgesics and let this group of patients suffer?

### 4.3. Characteristics of Selected Sedative and Analgesic Drugs That Are Potentially Useful during Cardiac Arrest and CPR

Choosing sedatives and analgesics that can be safely used in a patient undergoing CPR is challenging. The desirable characteristics include, but are not limited to, the support of myocardial and cerebral blood flow, rapid onset of action, and the ease of administration and storage. Medications that are most often used in local management protocols are ketamine, midazolam and fentanyl, or morphine [46].

Ketamine is a nonbarbiturate, phencyclidine derivative that produces sedation, amnesia, and, importantly, analgesia. Its action is a result of N-methyl-D-aspartate (NMDA) receptor antagonism as well as the interaction with opioid and cholinergic transmission. Ketamine is a sympathomimetic and it elevates the heart rate and blood pressure through sympathetic stimulation. This sympathomimetic effect of ketamine may help to facilitate the recovery of systemic blood pressure during cardiopulmonary resuscitation. In selected patients in whom significant elevations would be deleterious, the concurrent use of benzodiazepines or the administration of ketamine as a continuous infusion may be appropriate. In several animal studies of cardiac arrest, NMDA receptor antagonism was neuroprotective and positively influenced survival rates [46,47].

Midazolam is a benzodiazepine used for several indications in the hospital settings, including the induction of general anesthesia, pre-operative sedation, anxiolysis, and amnesia. Its cardiovascular profile is less encouraging than ketamine’s. Co-administration of midazolam with ketamine theoretically offers the benefit of preventing increases in the myocardial oxygen demand caused by the administration of ketamine [46].

Morphine and fentanyl belong to opioids, potent analgesics. These are substances that bind to opioid receptors, primarily mu-, kappa-, and delta-receptors, exerting therapeutic effects as well as adverse ones. Some preclinical studies have demonstrated that pre-treatment with opioids can preserve cellular integrity after acute hypoxia in many organs and tissues such as the brain, myocardium, or intestines. Two retrospective studies have shown that patients receiving opioids before or during cardiac arrest had statistically significantly higher survival rates and better neurological outcomes compared to untreated patients [47,48]. Fentanyl is considered hemodynamically neutral whereas morphine can cause hypotension through peripheral vasodilation [49,50].

## 5. Limitations of the Review

In none of the studies dealing with rib and/or sternal fractures was the outcome of the resuscitation reported—whether the patient’s circulation was restored could have significantly influenced subsequent decisions regarding the possible administration of analgesics. Additionally, none of these studies reported whether the patient received any analgesics in the peri-resuscitation period. It is currently impossible to give simple solutions to overcome these limitations. We think that future investigations should focus more on the use of analgesics in the immediate post-resuscitation period, especially for the survivors of prolonged resuscitative efforts.

## 6. Conclusions

Due to the small availability of studies that have dealt with the return of consciousness associated with CPR, it is not possible to clearly determine how often this occurs. Clearly more studies deal with chest trauma during resuscitation, but no study considered the use of analgesics. We noted a lack of standardized therapeutic approaches, which is probably due to the lack of guidelines for analgesic management during CPR. It is not surprising though, as most analgesics will precipitate the drop in systemic vascular resistance at a time when it is needed most, with the exception of ketamine.

To our knowledge, this is the first and only rapid review on the use of analgesics during resuscitative efforts, combining two instances in which pain control might be beneficial, i.e., the return of consciousness during CPR and chest injuries sustained by the cardiac arrest victims during chest compressions.

## 7. Implications for Clinical Practice

Currently, there are no guidelines for CPRIC management, so the decision to use analgesia and sedation is based on the experience of the resuscitation team and clinical perception. With the incidence of rib and sternum fractures differing from paper to paper, but still being significant, there seems to be a need for a systematic diagnosis of post-resuscitation injuries using imaging techniques (CT). which are more sensitive than X-rays. It would seem that the administration of analgesics should be considered in patients with a return of spontaneous circulation in whom resuscitation efforts have lasted a long time or during which mechanical chest compressions were used, leading to an increased incidence of rib, sternum, or internal organ injuries. There are no generally accepted guidelines as to the type and doses of sedative and analgesic drugs that could be used during resuscitation in patients with cardiac arrest. However, there are local management protocols in which the most commonly used drugs are ketamine, fentanyl, and midazolam [35].

## Figures and Tables

**Table 1:** PCC framework, inclusion and exclusion criteria, and search strategies.

	Inclusion Criteria	Exclusion Criteria
Participants (P)	Adults: emergency and ICU patients	Children (>18 years) non-ICU patients
Concept (C)	Chest compression, chest injuries, rib fractures, sternum fracture, and CPR-induced consciousness	No CPR
Context (C)	Cardiopulmonary resuscitation and cardiac arrest	Other diseases
Types of evidence source	Single-case report, cases report, letters to the editor, observational, prospective, and retrospective studies	Other studies
Years considered/Time period	All evidence published in the last 10 years, period 2010–2022	Publications prior to 2010
Language	English	Other languages
Databases	MEDLINE (PubMed), Web of Science, and Cochrane Library	Other databases
Key words	“Emergency Care”, “ICU”, “Cardiopulmonary resuscitation”, “Cardiac arrest”, “Chest compression”, „Chest injuries”, “Rib fractures”, “Sternum fracture”, and “CPR induced consciousness”	n/a

n/a—not applicable.

**Table 2:** Return of consciousness during cardiopulmonary resuscitation.

First Author, Year	The Resuscitation Site	Population	Country	Age	Gender	Arrest Rhythm	CPR Type	Analgesic/Sedation/Muscle Relaxant	Successful CPR	Signs of Return of Awareness during CPR
Pinto et al., 2020 [4]	ED	1	Portugal	89	Male	VF	Manual	Sedation was considered but was not performed.	No	The patient lower limb movements, tried to push away the CPR provider with his hands, bit endotracheal tube, showed facial signs of pain, and tried to move sideways.
Rice et al., 2016 [7]	ED	1	USA	55	Male	VF	Mechanical	Ketamine	Yes	Awake, alert, able to speak, purposeful movements during chest compressions
Greb et al., 2014 [15]	OH	1	USA	61	Male	VF	Manual and mechanical	No information	Yes	Agonal breathing, arm movements, and alertness.
Ulrichs et al., 2014 [16]	IH	1	Germany	24	Female	VF	n/d	Any sedative drugs	Yes	Able to recall specific and detailed descriptions of conversation CPR scene.
Sukumar, 2019 [17]	ED	1	India	52		N/D	Manual	No	Yes	Opening eyes, agitation, resisting the rescuer with his hands and head movements. No response to verbal commands.
Olaussen et al., 2015 [18]	n/d	10	Australia	M = 49.5	Male	VF, PEA, Asystole, and VT	Manual and mechanical	Sedation used but not recorded	n/d	Clinicians reported talking and reassuring the patient.
Oksar et al., 2016 [19]	IH	1	Turkey	69	Male	VF	Manual	No	Yes	Motor reflexes to painful stimuli.
Olaussen et al., 2017 [20]	n/d	112	Australia	N/D	N/D	N/D	Manual and mechanical	Morphine, fentanyl, midazolam, pancuronium, and suxamethonium	Yes and No	Eye opening, limb or body movement, and combativeness/
Pound et al., 2017 [21]	OH	1	Canada	59	Male	VF	Manual	Midazolam	Yes	Attempt to interfere or stop CPR and difficult intubation.
Grandi et al., 2017 [22]	ED	6	Italy	M = 67.5	Male	VF, PEA, Asystole, and VT	Manual and mechanical	Fentanyl, propofol, and rocuronium	Yes and No	Purposeful movements, open eyes, scream, respiratory movements, and ocular movements.
Doan TN et al., 2020 [23]	OH	52	Australia	M = 60	Male	Most initial shockable rhythm	Manual	Midazolam (0.5–2.5 mg iv. (given to four patients, either alone or in combination with fentanyl 25 mcg iv.), morphine 2.5 mg iv. (one patient), and ketamine 50 mg iv. in combination with suxamethonium 150 mg iv. (one patient).	Yes and No	Combativeness/agitation, groaning, and eye opening/ rolling.

OH—out-of-hospital; IH—intra-hospital ED—emergency department; n/d—no data; iv—intravenous; VF = ventricular fibrillation; PEA = pulseless electrical activity; VT = ventricular tachycardia; n/d—no data; and M—me.

**Table 3:** Chest injuries during CPR.

First Author, Year	Population	The Resuscitation Site	Age		Gender		CPR Type	Analgesic/Sedation/Muscle Relaxant	Rib Fractures (%)		Sternal Fractures (%)		Diagnostic Method
Kim et al., 2013 [24]	71	OH and IH	65		45M/26F		Manual	No information	22 (31)		3 (4)		CT
Hellevuo et al., 2013 [25]	170	IH	72		110M/60F		Manual	No information	41/153 (26.8)		16/153 (10.5)		Radiography, CT, and autopsy
Boland et al., 2014 [26]	235	OH	64		145M/90F		Manual and mechanical	No information	22 (9)		2 (1)		CXR CT
Smekal et al., 2014 [27]	222 †	OH	66.3 67.7		55M/28F 97M/42F		83 manual and 139 mechanical	No information	53 (64.6) 108 (78.8)		45 (54.2) 81 (58.3)		Autopsy
Kashiwagi et al., 2015 [28]	223	OH	75		129M/94F		Manual	No information	156 (69.96)		18 (8.07)		CT PMCT
Kralj et al., 2015 [29]	2148 †	OH and IH	61.8M 71.0F		1480M 668F		Manual and mechanical	No information	1140 (77) M 568 (85) F		878 (59) M 525 (79) F		Autopsy
Radinská et al., 2016 [30]	80 †	OH	58.2		61M/19F		Manual	No information	59 (73.7)		53 (66.3)		Autopsy
Yamaguchi et al., 2017 [31]	180 †	OH and IH	62		119M 61F		Most manual	No information	119 (66.1)		95 (52.8)		PMCT and autopsy
Dunham et al., 2017 [32]	39	OH	51.8		26M/13F		Manual	No information	33 (85%)		5 (13%)		CT
Beom et al., 2017 [33]	185	OH and IH	Before 2010	After 2010	Before 2010	After 2010	Manual	No information	Before 2010	After 2010	Before 2010	After 2010	MDCT
			63.2	62.6	21M/22F	89M/53F			27 (62.8)	112 (78.9)	13 (30,2)	38 (26.8)	
Yusufoglu et al., 2018 [34]	83	IH	65.67	70.19	34M/18F	14M/17F	Manual	No information	34 (65.4)	20 (64.5)	7 (13.5)	4 (12.9)	CT
Setälä et al., 2018 [35]	149 †	OH	70		56M/14F		Manual	No information	64 (43)		22 (15)		Autopsy
Friberg et al., 2019 [36]	414 †	OH and IH	66 68		44M/8F 244M/121F		52 manual and 365 mechanical	No information	40 (77) 349 (96)		20 (38) 291 (80)		Autopsy
Deliliga et al., 2019 [37]	88 †	OH and IH	60.6		53M/35F		Manual	No information	23 (26.1)		15 (17.4)		Autopsy
Kim et al., 2020 [38]	274	OH and IH	62.6		180M/94F		Manual	No information	182 (66)		32 (12)		CT
Jang SJ et al., 2020 [39]	43	IH	72.3		27M/16F		Manual	No information	43 (100)		31 (72.1)		CT
Zaidi et al., 2020 [40]	137	OH	62		63M/74F		Manual	No information	40 (29.2)				CT
Hamanaka et al., 2020 [41]	62 †	n/d	60		39M/23F		n/d	No information	40 (65)		n/d		Autopsy and PMCT
Prins et al., 2021 [42]	344	OH	66		259M/85F		Manual and mechanical	No information	285 (82.9)		98 (28.5)		CT
Canakci et al., 2021 [43]	178	IH	73.0 71.0		66M/65F 33M/14F		131 manual and 47 mechanical	No information	14 (10.7) 3 (6.4)		1 (0.6) 0 (0)		CT
Takayama et al., 2018 [44]	472	OH	77.6		374M/98F		Manual	No information	233 (49.4) *				CT
									180 (38)		1 (0.2)		

† deceased during CPR or after hospital admission. * skeletal chest injuries. OH—out-of-hospital; IH—intra-hospital ED—emergency department; n/d—no data; iv—intravenous; VF = ventricular fibrillation; PEA = pulseless electrical activity; VT = ventricular tachycardia; n/d—no data; and M—me.

## Data Availability

The authors declare that the data of this research are available from the correspondence author on request.
